# Current Advances in Allosteric Modulation of Muscarinic Receptors

**DOI:** 10.3390/biom10020325

**Published:** 2020-02-18

**Authors:** Jan Jakubik, Esam E. El-Fakahany

**Affiliations:** 1 Department of Neurochemistry, Institute of Physiology CAS, 142 20 Prague, Czech Republic; 2 Department of Experimental and Clinical Pharmacology, University of Minnesota College of Pharmacy, Minneapolis, MN 55455, USA

**Keywords:** acetylcholine, muscarinic receptors, allosteric modulation

## Abstract

Allosteric modulators are ligands that bind to a site on the receptor that is spatially separated from the orthosteric binding site for the endogenous neurotransmitter. Allosteric modulators modulate the binding affinity, potency, and efficacy of orthosteric ligands. Muscarinic acetylcholine receptors are prototypical allosterically-modulated G-protein-coupled receptors. They are a potential therapeutic target for the treatment of psychiatric, neurologic, and internal diseases like schizophrenia, Alzheimer’s disease, Huntington disease, type 2 diabetes, or chronic pulmonary obstruction. Here, we reviewed the progress made during the last decade in our understanding of their mechanisms of binding, allosteric modulation, and in vivo actions in order to understand the translational impact of studying this important class of pharmacological agents. We overviewed newly developed allosteric modulators of muscarinic receptors as well as new spin-off ideas like bitopic ligands combining allosteric and orthosteric moieties and photo-switchable ligands based on bitopic agents.

## 1. Introduction

Slow metabotropic responses to acetylcholine are mediated by muscarinic receptors. Five distinct subtypes of muscarinic acetylcholine receptors (M_1_–M_5_) have been identified in the human genome [1]. The structure of all five receptor subtypes was resolved by X-ray crystallography [2,3,4,5,6]. Muscarinic receptors are members of class A of the G-protein-coupled receptor (GPCR). M_1_, M_3_, and M_5_ subtypes preferentially activate phospholipase C and calcium mobilization through G_q/11_, whereas M_2_ and M_4_ receptors inhibit the activity of adenylyl cyclase by activation of the α-subunit of the G_i/o_ family of G-proteins. The latter two receptors also modulate the conductance of ion channels (e.g., inward rectifying potassium ion channels) by βγ-dimers of the G_i/o_ G-proteins [7]. Muscarinic receptors mediate a wide range of physiological functions in the central and peripheral nervous system and innervated tissues. Muscarinic receptors thus represent a potential therapeutic target for the treatment of psychiatric and neurologic conditions (e.g., schizophrenia, Alzheimer’s disease, Huntington disease) [8,9] as well as internal diseases (e.g., type 2 diabetes, asthma, chronic pulmonary obstruction, incontinence) [10,11,12].

**Figure 1:**
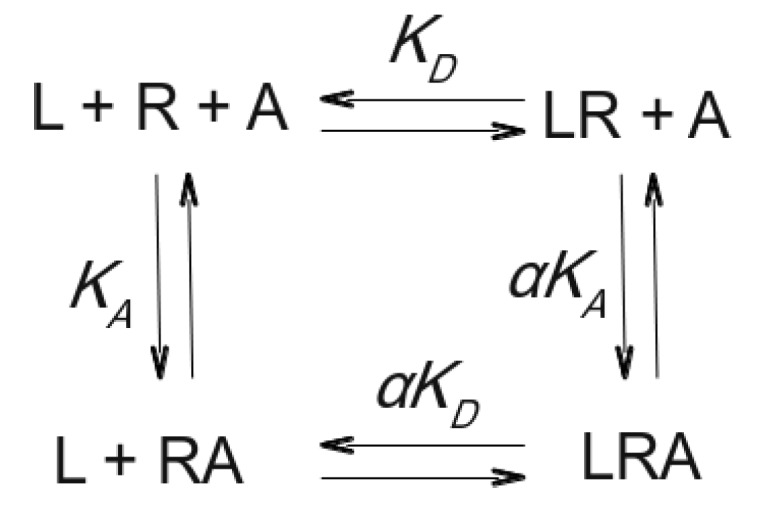
An orthosteric ligand L binds to the receptor R with equilibrium dissociation constant K_D_, and an allosteric modulator A binds to the receptor R with an equilibrium dissociation constant K_A_. The orthosteric ligand L and the allosteric modulator A can bind concurrently to the receptor R to form a ternary complex LRA. Binding of one ligand to the receptor changes the equilibrium dissociation constant of the other ligand by a factor of cooperativity α [15].

The concept of allosterism was formally introduced into the field of enzymology by Monod et al. [13] and Koshland et al. [14] in 1965 and 1966, respectively. The former model was termed concerted, the latter one sequential. Allosteric modulation of GPCR is much simpler than that of enzymes. GPCR allosteric modulators bind to a site on the receptor that is spatially distinct from that of the endogenous transmitter, acetylcholine, in the case of muscarinic receptors. Consequently, binding of an allosteric modulator and an orthosteric ligand is not mutually exclusive, i.e., both ligands may bind to the receptor simultaneously to form a ternary complex (Figure 1). Binding of allosteric modulators induces a change in the conformation of the receptor that results in changes in affinity (eventually potency and efficacy) of the orthosteric ligand [15].

Based on the effects of an allosteric modulator on the affinity of an orthosteric ligand, allosteric modulators may be classified into three categories: 1. Positive allosteric modulators (PAM) that increase the affinity of orthosteric ligands; 2. Negative allosteric modulators (NAM) that decrease the affinity of orthosteric ligands; and 3. Neutral allosteric modulators that do not affect the affinity of the orthosteric ligand. When the intrinsic efficacy of allosteric modulator is taken into account, these three categories expand to six: 1. Pure PAMs; 2. PAM-agonists that possess intrinsic agonistic propensity in the absence of the orthosteric agonists they modulate; 3. PAM-antagonists that lower the efficacy of the agonists they modulate [16]; 4. Pure NAMs; 5. NAM-agonists that possess own agonistic propensity in the absence of the agonists and activate the receptor, while they negatively modulate endogenous agonist [17]; 6. Silent allosteric modulators (SAMs) that, although they bind to the receptor, do not affect the affinity, potency, or efficacy of the orthosteric ligand and do not have agonistic propensity on their own. In interaction with agonist, an allosteric modulator may affect both agonist affinity and efficacy. Thus, each of the six abovementioned categories has three sub-categories based on positive, negative, or neutral effects (cooperativity) of the allosteric modulator on agonist efficacy. However, six basic categories are sufficient for the general classification.

As early as in 1969, Lüllmann et al. showed in their pioneering work that alkane-bis-ammonium compounds inhibited the functional response to the conventional muscarinic agonist carbachol non-competitively [18]. Later, Clark and Mitchelson discovered that gallamine similarly inhibited the action of acetylcholine and carbachol on the function of heart atria in a non-competitive manner [19]. The concentration-response curves to the agonists were shifted to the right, but the magnitude of the progressive shifts diminished with increasing concentrations of gallamine. When the action of acetylcholine on the heart was evaluated in the combined presence of gallamine and the antagonist atropine, the inhibition of functional response to carbachol was smaller than expected for the effects of two competitive antagonists. These observations led to the conclusion that the action of gallamine takes place at an allosteric site on the receptor, resulting in negative cooperativity with the binding of both orthosteric agonists and antagonists. Since then, a wide variety of allosteric modulators has been discovered. These include inhibitors of acetylcholinesterase, ion channel blockers, various alkaloids, small peptides, etc. For review, see [20]. Thanks to early intensive research, muscarinic receptors became a useful prototype of allosterically-modulated GPCRs.

## 2. Advantages of Allosteric Modulators as Therapeutics

### 2.1. Selectivity by Targeting Less Conserved Domains on the Receptor 

Muscarinic receptor subtypes share high structural homology in the transmembrane domains where the orthosteric binding site is located. On the other hand, domains out of the membrane are less conserved. Targeting allosteric domains allows achieving binding selectivity for certain receptor subtypes to the extent which is not possible with orthosteric ligands.

### 2.2. Conservation of Space and Time Pattern of Signaling

Theoretically, a pure PAM of acetylcholine would only induce an action when endogenous acetylcholine is released. Consequently, its action would be restricted in space and time to those synapses where signaling is currently happening. Thus, space and time patterns of signaling could be restored under diminished acetylcholine release that is typical in neurodegenerative disorders, e.g., Alzheimer’s disease. Successful action of PAMs requires functional post-synaptic receptors. Encouragingly, post-synaptic receptors are relatively intact in neurodegenerative diseases like Alzheimer’s disease or senile dementia of Lewy body type [21,22].

### 2.3. Absolute Selectivity

In practice, absolute selectivity due to exclusive binding to a single receptor subtype is hard to achieve. Alternatively, the absolute selectivity of an allosteric agent can be achieved by having a ligand with the desired cooperativity at a given receptor subtype and neutral (silent) cooperativity at the rest of the receptor subtypes. Selectivity may be derived from binding cooperativity as well as from effects on potency and efficacy.

### 2.4. Selective Blocking of Activated Receptors

PAM-antagonists that exhibit partial or absolute selectivity for a given receptor subtype may be used to selectively target receptors activated by the endogenous neurotransmitter located in specific tissues or organs [16]. This feature can be used to selectively reverse persistent excessive agonism under certain pathological conditions (e.g., bronchospasm in asthma [23]) or overstimulation of salivary and lacrimal glands after organophosphate poisoning in case of M_3_-selective PAM-antagonists.

## 3. Location of the Allosteric Binding Sites on Muscarinic Receptors

**Figure 2:**
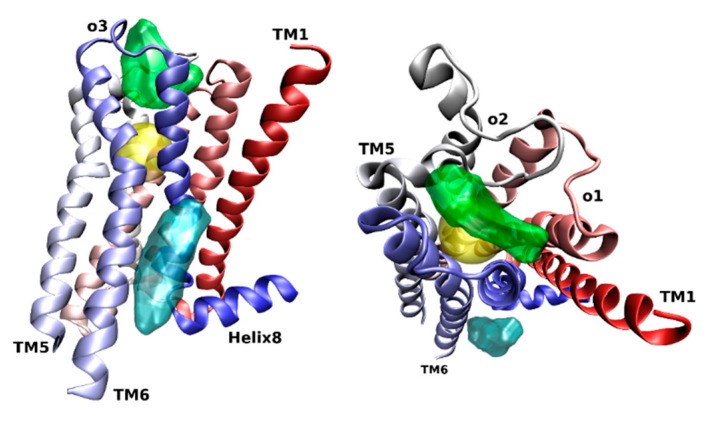
Side view with TM6 front (left), and extracellular view (right) of the orthosteric (yellow), common allosteric (green), and cholesterol (cyan) binding sites at the M_2_ receptor (4MQT) (colored in red-white-blue gradient).

The binding site of classical allosteric modulators like gallamine, alcuronium, or alkane-bis-ammonium compounds has been located between the second (o2) and third extracellular (o3) loops (Figure 2, green) [24,25,26]. The charged EDGE motif in the o2 loop plays a critical role in the binding of these ligands [27]. In the crystal structure of the M_2_ receptor [4], the allosteric modulator LY2119620 (that binds to the same site between o2 and o3) does not form a hydrogen bond to E172 or E175 of the EDGE motif but makes π–π interactions to the adjacent Y177 in the o2 and Y426 in the extracellular edge of TM7.

**Figure 3:**
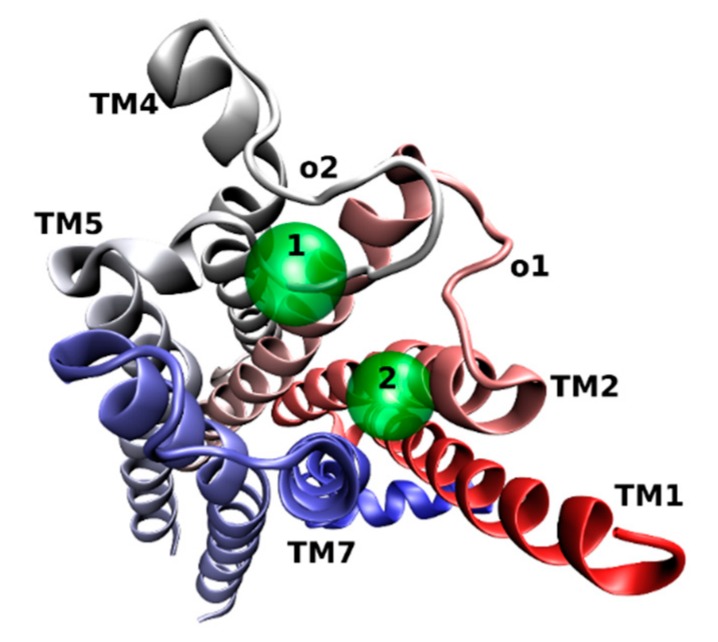
Extracellular view of two binding centers (green) in the common allosteric binding site at the M_2_ receptor (4MQT) (colored in red-white-blue gradient).

**Figure 4:**
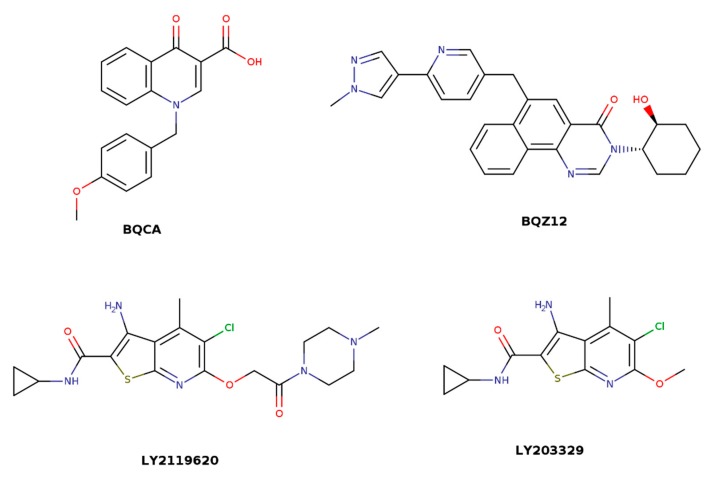
Structures of M_1_-selective benzyl quinolone carboxylic acid (BQCA), BQZ12, M_2_-selective LY2119620, and M_4_-selective LY2033298 allosteric modulator.

Computer modeling studies of allosteric ligand binding to M_2_ receptors have revealed two centers for binding of the electropositive part of allosteric ligands (Figure 3) [28]. The first center consists of Y177, N410, N419, and W422, and the second consists of Y80, Y83, T84, and T423. Further, this study showed that alkane-bis-ammonium compounds, gallamine, alcuronium, and strychnine bind to the fist center. M_1_-selective PAM benzyl quinolone carboxylic acid (BQCA, Figure 4) binds to Y179 and F182 in the o2 loop, and E397 and W400 in TM7 [29]. Thus, the binding site of BQCA overlaps with the common allosteric binding site. In contrast, M_4_-selective PAM LY2033298 (Figure 4) interacts with the o1, o2, and o3 loops [30]. Key binding residues of LY2033298 are K95 in the o1, F186 in o2, and D432 in o3 (M_4_ numbering). A cryptic allosteric binding pocket in the extracellular domain that is absent in existing crystal structures has been predicted by simulation of molecular dynamics and confirmed by mutagenesis experiments [31]. This cryptic pocket is dynamically formed in the vicinity of the common allosteric site center 1 by rearrangement of conserved E^7.36^ in TM7, Y^2.64^ in the o2 loop close to TM2, and conserved C^45.50^ in the middle of the o3 loop (general GPCR numbering [32,33]). The site is preferentially formed at the M_1_ receptor and has been identified as a binding site of highly M_1_-selective PAM BQZ12 (Figure 4) [34,35].

While the majority of known muscarinic allosteric ligands bind to the site between the o2 and o3 loops, sterol-based WIN-compounds have been found to interact with gallamine and strychnine in a non-competitive manner [36]. Thus, the binding site for WIN-compounds is not between the o2 and o3 loops but somewhere else. However, the precise location of the WIN-compound binding site has not been determined yet. Interestingly, cholesterol also allosterically modulates the binding and function of muscarinic receptors [37,38,39,40]. Using site-directed mutagenesis, the binding site for membrane cholesterol has been located to the groove between TM6 and TM7 in the intracellular leaflet of the membrane [40]. Membrane cholesterol allosterically modulates many GPCRs [41]. Cholesterol has co-crystallized with GPCRs at various sites, both in the extracellular and intracellular leaflet of the membrane [41]. Computer modeling of the interaction between membrane cholesterol and GPCR suggests the possibility of several cholesterol binding sites per one molecule of GPCR [42]. How many cholesterol binding sites muscarinic receptors do have and whether sterol-based WIN-compounds bind to the cholesterol-binding site remains to be elucidated.

## 4. Molecular Mechanisms of Action of Allosteric Modulators

The maximum magnitude of the effects of an allosteric modulator at a given receptor (binding cooperativity) varies from one orthosteric ligand to another. Comparison of the binding cooperativity of a given allosteric ligand with several orthosteric ligands has suggested that cooperativity is dependent on the distance between the electropositive and electronegative part of the orthosteric ligand [43]. Thus, data has suggested that allosteric modulators change the distance between TM3 interacting with electropositive and TM6 interacting with an electronegative part of the orthosteric ligand. This hypothesis was confirmed later by crystal structures and molecular modeling [4,44]. Numerous studies have identified differential key amino acids to govern allosteric action of various allosteric modulators, suggesting the existence of multiple allosteric switches on muscarinic receptors. For example, M_4_-selective PAM LY2033298 gains its efficacy from interaction with K95 in the o1 loop, and its binding cooperativity results from interaction with F186 in the o2 loop (M_4_ numbering) [30]. In contrast, tyrosine in the o2 loop (Y179 in M_1_, Y177 in M_2_) and tryptophan in TM7 (W^7.35^, Ballesteros-Weinstein numbering [45]) have been identified as key residues, defining the magnitude of binding cooperativity of M_1_-selective PAM of acetylcholine (BQCA) as well as M_2_-selective PAM of iperoxo (LY2119620, Figure 4) [4,29,34]. The common feature found for all PAMs is shrinkage of the vestibule to the orthosteric binding site that is accompanied by the closure of the binding pocket [46]. Divergent mechanisms underlying how shrinkage of the vestibule is achieved probably represent the molecular basis of PAM receptor subtype selectivity.

**Figure 5:**
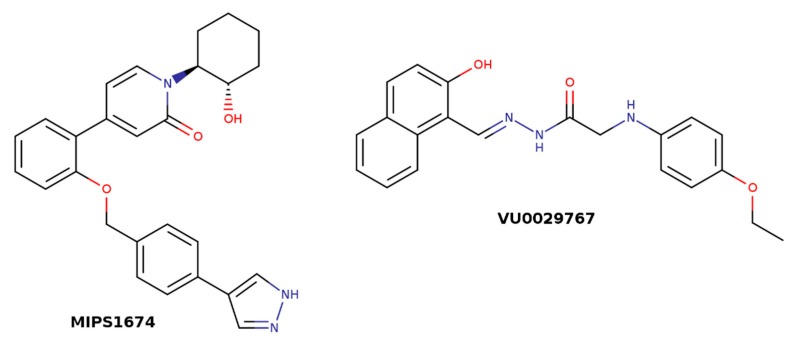
Structures of M_1_ allosteric modulators MIPS1674 and VU0029767.

Many GPCRs, including muscarinic receptors, activate several signaling pathways upon activation by agonists [47,48,49]. It is generally accepted that structurally different agonists induce specific changes in the conformation of GPCRs that can lead to non-uniform modulation of signaling pathways. This preferential orientation of the signaling of a GPCR towards a subset of its signal transducers is termed signaling bias [50]. In principle, the structure of the receptor in a ternary complex with agonist and the allosteric modulator is distinct from the one in a binary complex with agonist only and may result in biased signaling too. Attaining signaling bias via allosteric modulation could bring a new plethora of possibilities to modulate receptor function. It has been shown at M_1_ receptors that the allosteric modulator VU0029767 (Figure 5) acts as a strong PAM of acetylcholine-induced intracellular calcium mobilization while its effects on acetylcholine-induced activation of phospholipase D were marginal [51]. Based on this observation, it has been suggested that differential modulation of receptor coupling to downstream signaling pathways by allosteric modulators may result in signaling bias. However, structurally divergent M_1_ PAMs BQCA (Figure 4) and MIPS1674 (Figure 5) have shown only small differences in the modulation of the inositol phosphate, β-arrestin, and ERK_1/2_ pathways [52]. Residues near the common allosteric binding site have been implicated in mediating biased signaling even of orthosteric ligands at several GPCRs [53]. Thus, divergent mechanisms of shrinkage of the vestibule to the binding pocket may not only stand behind subtype-selective effects of allosteric modulators but also drive ligand bias that is common to the entire A class of GPCRs [54].

## 5. Role of the Common Allosteric Binding Site in the Binding of Orthosteric Ligands

The common allosteric binding site located between the o2 and o3 loops represents the vestibule to the orthosteric binding site. The model in which an orthosteric ligand binds transiently to a secondary site before moving to the orthosteric binding site (so-called tandem two-site model [55]) has been proposed to explain apparent receptor isomerization upon antagonist binding [56,57]. Interaction of orthosteric ligands with the allosteric domain between the o2 and o3 loops has been confirmed and studied in detail by computer modeling [3,58,59]. These studies showed that two-step binding is common for all orthosteric ligands studied and that orthosteric ligands interact primarily with Y177 (common allosteric center 1), N410, N419, and W422 (common allosteric center 2) (M_2_ numbering). Subtype variations of affinity and kinetics of orthosteric ligands may be attributed to variation in their interaction with the allosteric site [6].

The strong interaction of tiotropium with the allosteric binding site also explains the discrepancy between extremely slow binding kinetics and immediate inhibitory action of this bronchodilator [60]. Tiotropium binding to the allosteric site is fast and results in antagonism of the functional effects of the M_3_ receptor.

The M_2_-selective antagonist methoctramine competitively inhibits the binding of orthosteric ligands. High concentrations slow down the dissociation of [^3^H]N-methylscopolamine. Detailed analysis of methoctramine binding has revealed two modes of interaction with the receptor. Transient binding to the common allosteric site center 1 (namely E175 in the o2 loop) is followed by stable binding that occurs concurrently to both the allosteric and orthosteric sites [61]. Methoctramine is thus a bitopic dualsteric antagonist.

## 6. Bitopic Ligands

**Figure 6:**
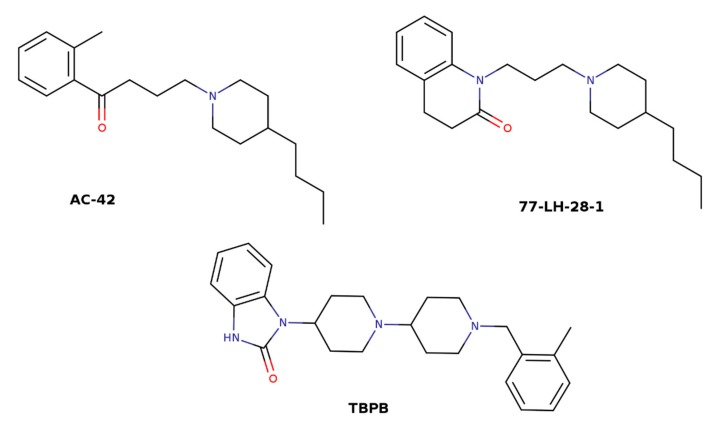
Structures of bitopic ligands AC-42, 77-LH-28-1, and TBPB.

The idea of utilizing high efficacy of orthosteric ligands and subtype diversity of the allosteric binding sites has led to the development of bitopic ligands that span both sites and interact with them concurrently. In principle, a bitopic ligand consists of two molecules, one targeting the orthosteric site, and the other interacting with the allosteric site. The two parts are connected by a linker of a proper length. A combination of the nonselective muscarinic agonist iperoxo and its M_1_-selective PAM BQCA has led to a set of M_1_-selective agonists with graded efficacy that is dependent on the chemical structure of the linker [62]. The orientation of the orthosteric moiety within the orthosteric binding site is crucial for subtype selectivity [63]. Therefore, the structure of the linker affects not only efficacy but also the selectivity of bitopic ligands. Another example of a bitopic agonist that gains its selectivity from interaction with the common allosteric binding site is TBPB (Figure 6) [64,65]. Mutations of the allosteric and orthosteric binding sites have revealed that NDMC (N-desmethylclozapine) that is considered an allosteric agonist and McN-A-343 (4-(m-chlorophenyl-carbamoyloxy)-2-butynyltri-methylammonium) that is considered an orthosteric agonist are in fact bitopic ligands [66,67]. Allosteric effects of the bitopic agonists McN-A-343 and NDMC and the allosteric agonists AC-42 and 77-LH-28-1 (Figure 6) are mediated via Y177 in the o2 loop of the M_2_ receptor [66]. Taken together, these bitopic ligands also interact with the center 1 (Figure 3) of the common allosteric binding site (Figure 2).

**Figure 7:**
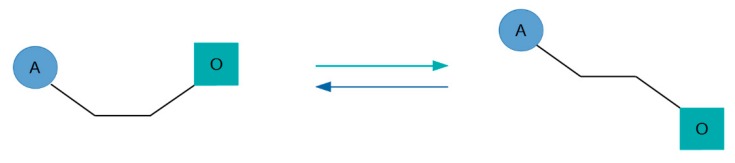
Scheme of isomerization of photo-switchable ligand, consisting of the orthosteric (O) and allosteric (A) moiety connected by the linker.

Further development of bitopic ligands has led to the discovery of the photo-switchable ligands [68]. In the case of a photo-switchable ligand, the conformation of the linker is sensitive to exposure to light of a specific wavelength, which leads to the isomerization of the linker and change in the mutual orientation of orthosteric and allosteric moieties (Figure 7). In one linker conformation, the orthosteric and allosteric moieties are in an orientation that does not allow simultaneous interaction of individual moieties with their respective binding sites that makes a ligand inactive. After linker isomerization, the orientation of individual moieties allows simultaneous interaction of individual moieties with their respective binding sites, and the ligand becomes active. Photo-switchable ligands are an invaluable tool in basic research since they allow an experimenter to apply ligand at the desired place and desired time in the blink of a laser. 

Besides being an interesting research tool, bitopic muscarinic ligands, consisting of the orthosteric and allosteric moiety, have also a translational potential. For example, bis(ammonio)alkane-type compounds (hybrid molecules of the orthosteric agonist iperoxo and allosteric moiety derived from naphmethonium or W84) can be used as non-opioid non-steroidal analgesics [69] or inhibitors of cell growth for glioblastoma therapy [70].

## 7. Novel Allosteric Modulators

Although positive allosteric modulation of acetylcholine was presented as proof of concept in the 1990s [71,72], it took more than 10 years to develop first PAMs with physiologically active properties suitable for further drug development [73,74,75,76]. However, the progress of research on muscarinic allosteric modulators has accelerated, and selective PAMs for each subtype have been discovered during the last decade.

**Figure 8:**
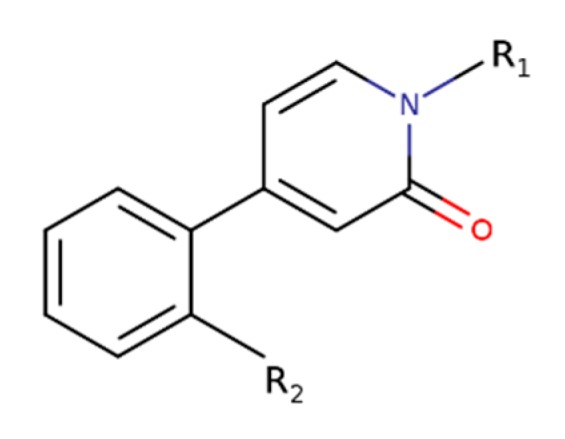
The core structure of 4-phenylpyridin-2-one derivatives.

Namely, two new classes of M_1_-selective PAMs have been developed. Derivatives of heterocyclic carboxamides display various degrees of intrinsic activity and various modulatory profiles of affinity and efficacy of acetylcholine [77]. Similarly, derivatives of 4-phenylpyridin-2-one (Figure 8) display a diverse range of activities, ranging from pure PAMs to pure allosteric agonists [78]. All 4-phenylpyridin-2-one derivatives have retained exquisite selectivity for the M_1_ receptor.

The availability of the M_2_ receptor crystal structure in an inactive [2], as well as active conformation [4] and advances in computer modeling, has allowed for the acceleration of structure-based design of chemically diverse allosteric modulators of this receptor [79]. The combination of in silico simulation of molecular dynamics and virtual screening has led to the identification of allosteric modulators of the M_2_ receptor of chemically novel structures that have been verified as NAMs and PAMs of superagonist iperoxo in binding experiments.

Positive allosteric modulators of antagonists may turn otherwise a non-selective orthosteric antagonist to the subtype-selective antagonist with therapeutic potential. Recently, as a proof of concept, M_2_-selective positive modulators of orthosteric antagonists have been developed [80]. In this study, triazolo-quinazolinone analogs were identified as M_2_ selective positive modulators of N-methylscopolamine (NMS) by docking a large library of molecules to the allosteric binding site of the M_2_ receptor in an inactive conformation. Resulting allosteric modulators increased NMS affinity about 5-times and slowed NMS dissociation from the M_2_ receptor about 50-fold while having no such effects at the other subtypes of muscarinic receptors.

Testing of novel muscarinic allosteric modulators in animal models of human diseases has shown their good efficacy. Cholinergic neurons regulate glutamatergic neurons in the striatum that regulate selection and decision-making behavior [81]. The M_1_-selective PAM of acetylcholine BQCA has improved efficacy of the antipsychotics haloperidol, clozapine, and aripiprazole in the glutamatergic deficit mouse model of behavior [82]. M_1_ receptors play a critical role in cognitive processes [7]. Thus, positive allosteric modulation of M_1_ receptors appears to be the way to treat cognitive deficits in Alzheimer’s disease or schizophrenia [83]. However, cortex, hippocampus, and striatum post mortem samples from some patients with schizophrenia have shown a profound loss of muscarinic receptors and decreased responsiveness to M_1_-selective PAM of acetylcholine BQCA. These findings may explain why some individuals with schizophrenia may not respond to such [84].

The striatal M_4_ receptors attenuate dopaminergic and glutamatergic neurotransmission [85]. Thus, selective positive modulation of M_4_ receptors may provide a novel treatment strategy of psychotic symptoms in schizophrenia that are considered to result from dopaminergic hyperactivity [83]. A series of M_4_ PAMs have been found to be centrally active and efficient in an animal model of schizophrenia [86]. Huntington’s disease results from increased transmission at glutamatergic corticostriatal synapses that may be attenuated by the cholinergic system. In accordance, M_4_ PAMs have improved behavioral symptoms in the mouse model of Huntington disease [76]. 

**Figure 9:**
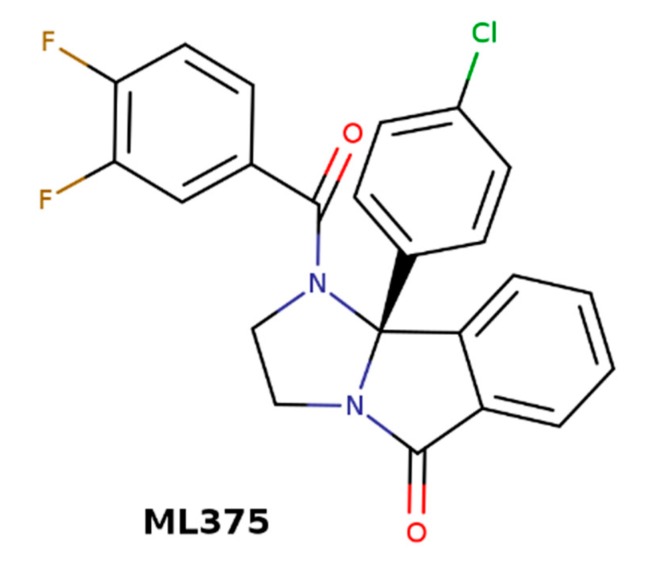
Structure of M_5_-selective negative allosteric modulators (NAM) of acetylcholine ML375.

M_5_ muscarinic receptors are expressed solely in the substantia nigra and ventral tegmental area (VTA) [7]. Cholinergic neurons in the nucleus accumbens (NAcc) project to VTA, where they stimulate dopaminergic neurons. The VTA dopaminergic neurons project back to NAcc, where they stimulate cholinergic neurons. This positive feedback works as the reward circuit [87]. A blockade or negative allosteric modulation of M_5_ receptors could break the reward cycle and prevent addiction. The M_5_-selective NAM of acetylcholine ML375 (Figure 9) [88] has effectively prevented cocaine self-administration and the development of addiction in rats [89].

**Figure 10:**
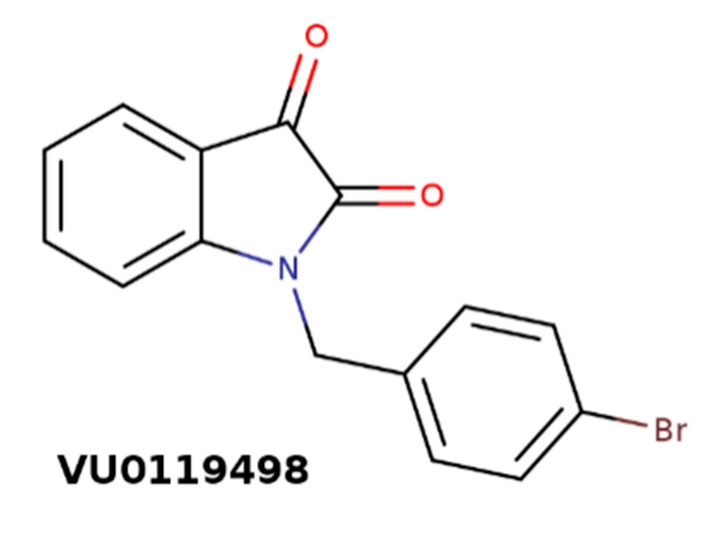
Structure of M_3_-selective positive allosteric modulators (PAM) of acetylcholine VU0119498.

In type 2 diabetes, pancreatic β cells are unable to release sufficient amounts of insulin to maintain physiological blood glucose levels. Parasympathetic activation of pancreatic β cells by acetylcholine stimulates the release of insulin [90]. Effects of acetylcholine on insulin secretion are mediated by the M_3_ receptor [91]. In accordance, M_3_ PAM of acetylcholine VU0119498 (Figure 10) has been shown to enhance glucose-stimulated insulin secretion and greatly improve glucose tolerance in lean and obese glucose-intolerant mice [92]. These effects were absent from mice lacking M_3_ receptors in their β cells.

## 8. Perspectives

Recent findings of the diversity of actions of allosteric modulators and bitopic ligands of muscarinic receptors have greatly influenced the development of new therapies for a variety of disorders. Recent discoveries of selective positive allosteric modulators of acetylcholine with therapeutic potential in the treatment of psychiatric and neurologic disorders like Alzheimer’s or schizophrenia, as well as internal disorders like type 2 diabetes, are encouraging. Research on several muscarinic allosteric modulators has surpassed the stage of optimization of selectivity, efficacy, and bioavailability and advanced to a translational stage of medicinal research. In the field of basic research, further progress is mainly achieved by the introduction of novel concepts and techniques. Novel photo-switchable ligands may greatly increase the accuracy of timely delivery as well as allow varied permutations of experimental design. Thus, focus on the design of novel photo-switchable ligands is currently gaining momentum.

## Nomenclature

**Compounds**	**Chemical Names**
**77-LH-28-1**	1-[3-(4-Butylpiperidino)propyl]-3,4-dihydroquinoline-2(1H)-one
**AC-42**	4-(4-Butylpiperidin-1-yl)-1-o-tolylbutan-1-one
**Alcuronium**	2-[(1S,9Z,11S,13S,17S,25Z,27S,33S,35S,36S)-38-(2-hydroxyethylidene)-14,30-bis(prop-2-enyl)-8,24-diaza-14,30-diazoniaundecacyclo [2 5.5.2.2^11,14^.1^1,26^.1^10,17^.0^2,7^.0^13,17^.0^18,23^.0^30,33^.0^8,35^.0^24,36^]octatriaconta-2,4,6,9,18,20,22,25-octaen-28-ylidene]ethanol
**BQCA**	1-(4-Methoxybenzyl)-4-oxo-1,4-dihydroquinoline-3-carboxylic acid
**BQZ12**	3-((1S,2S)-2-hydroxycyclohexyl)-6-((6-(1-methyl-1H-pyrazol-4-yl)pyridin-3-yl)methyl)benzo [h]quinazolin-4(3H)-one
**Gallamine**	2-[2,3-bis [2-(triethylazaniumyl)ethoxy]phenoxy]ethyl-triethylazanium
**LY2119620**	3-Amino-5-chloro-N-cyclopropyl-4-methyl-6-[2-(4-methylpiperazin-1-yl)-2-oxoethoxy]thieno [2,3-b]pyridine-2-carboxamide
**LY2033298**	3-Amino-5-Chloro-N-Cyclopropyl-6-Methoxy-4-Methylthieno [2,3-B]Pyridine-2-Carboxamide
**McN-A-343**	4-(m-chlorophenyl-carbamoyloxy)-2-butynyltri-methylammonium
**Methoctramine**	*N*,*N*′-bis [6-[[(2-methoxyphenyl)-methyl]hexyl]-1,8-octane]diamine
**MIPS1674**	1-[(1S,2S)-2-hydroxycyclohexyl]-4-[2-[[4-(1H-pyrazol-4-yl)phenyl]methoxy]phenyl]pyridin-2-one
**ML375**	(S)-9b-(4-chlorophenyl)-1-(3,4-difluorobenzoyl)-2,3- dihydro-1H-imidazo [2,1-a]isoindol-5(9bH)-one
**Naphmethonium**	[3-(1,3-dioxobenzo [de]isoquinolin-2-yl)-2,2-dimethylpropyl]-[6-[3-(1,3-dioxoisoindol-2-yl)propyl-dimethylazaniumyl]hexyl]-dimethylazanium
**NDMC**	N-desmethylclozapine; 3-chloro-6-piperazin-1-yl-11H-benzo [b] [1,4]benzodiazepine
**NMS**	N-methylscopolamine; [(1S,2S,4R,5R)-9,9-dimethyl-3-oxa-9-azoniatricyclo [3.3.1.0^2,4^]nonan-7-yl] (2S)-3-hydroxy-2-phenylpropanoate
**Strychnine**	(4aR,5aS,8aR,13aS,15aS,15bR)-4a,5,5a,7,8,13a,15,15a,15b,16-decahydro-2H-4,6-methanoindolo [3,2,1-ij]oxepino [2,3,4-de]pyrrolo [2,3-h]quinolin-14-one
**TBPB**	1-(1′-(2-methylbenzyl)-1,4′-bipiperidin-4-yl)-1H-benzo [d]imidazol-2(3H)-one
**Tiotropium**	[(1S,2S,4R,5R)-9,9-dimethyl-3-oxa-9-azoniatricyclo [3.3.1.02,4]nonan-7-yl]2-hydroxy-2,2-dithiophen-2-ylacetate
**VU0119498**	1-(4-bromobenzyl)indole-2,3-dione
**VU0029767**	(E)-2-(4-ethoxyphenylamino)-N′-((2-hydroxynaphthalen-1-yl)methylene)acetohydrazide
**W84**	6-[dimethyl-[3-(4-methyl-1,3-dioxoisoindol-2-yl)propyl]azaniumyl]hexyl-dimethyl-[3-(4-methyl-1,3-dioxoisoindol-2-yl)propyl]azanium
